# Magnesium Silicate Coatings Were Prepared by Micro-Arc Oxidation on the Surface of Magnesium Alloys Through the Synergistic Effect of SiO_3_^2^^−^/F^−^

**DOI:** 10.3390/ma18204760

**Published:** 2025-10-17

**Authors:** Yuru Zhang, Shudong Zhang, Hongtao Li, Cancan Liu, Hao Wang, Li Ma

**Affiliations:** 1 College of Materials Science and Engineering, Nanjing Tech University, Nanjing 211800, China; zhangyuru20002023@163.com (Y.Z.); 13476728993@163.com (H.W.); 17855040576@163.com (L.M.); 2 Department of EMC Equipment Testing, Jiangsu Institute of Medical Device Testing, No. 17, Kangwen Road, Nanjing 210019, China; zhang.83@163.com

**Keywords:** micro-arc oxidation, KF, SiO_3_^2^^−^/F^−^, phase composition

## Abstract

To enhance the long-term corrosion resistance of micro-arc oxidation coatings on magnesium alloys, this study regulated the ionic composition of the electrolyte based on the solubility product rule. In the silicate system, a micro-arc oxidation coating mainly composed of magnesium silicate was successfully prepared on AZ31A magnesium alloy by synergistically optimizing the ratio of SiO_3_^2^^−^/F^−^. The results show that the addition of KF significantly promotes coating growth, with the thickness increasing from 19.32 μm to a maximum of 46.86 μm. As the Na_2_SiO_3_ concentration increases, the main phase of the coating changes from MgO to Mg_2_SiO_4_. Electrochemical tests indicate that the coating prepared with 30 g/L Na_2_SiO_3_ and KF addition exhibits the best corrosion resistance, demonstrating the lowest corrosion current density of 3.89 × 10^−^^9^ A·cm^−^^2^, which is approximately four orders of magnitude lower than that of the uncoated substrate. However, when the Na_2_SiO_3_ concentration is too high, the corrosion resistance decreases due to increased pore size and defects, confirming a non-monotonic relationship between silicate concentration and coating performance.

## 1. Introduction

Magnesium is a lightweight metal with a density of only 1.74 g/cm^3^, approximately two-thirds that of aluminum and one-quarter that of steel [1]. As one of the lightest structural materials, magnesium alloys possess specific strength and stiffness, enabling them to withstand high loads while maintaining their weight advantage [2,3]. However, the absolute strength of magnesium alloys is lower than that of high-strength aluminum alloys and steel. Furthermore, their low electrode potential, along with poor wear and corrosion resistance, limits their wider application [4,5,6,7]. To address the issue of insufficient strength, heat treatment is one of the most effective strengthening methods, which primarily strengthens the matrix through the precipitation of fine second phases [8,9]. A reinforced substrate is crucial for supporting functional coatings. In addition, the inherent corrosion sensitivity of magnesium stems from its low electrode potential and the instability of its surface film. The latest insights into the corrosion mechanism indicate that microstructure factors have a significant impact on it, such as crystal orientation [10], and the harmful effect of chloride ions in destroying surface films at the atomic scale [11].

Therefore, surface treatment is necessary prior to industrial application. Micro-arc oxidation (MAO), an advanced surface modification technique evolved from conventional anodic oxidation, is widely employed in the treatment of magnesium alloys. This process enables the formation of a coating that is strongly adhered to the substrate and exhibits excellent resistance to corrosion and wear.

The corrosion resistance of micro-arc oxidation coatings is affected by multiple variables, mainly including electrical parameters and electrolytes [12,13,14,15,16]. Among them, electrical parameters have a relatively minor impact as external factors, while the electrolyte, as an internal factor, directly determines the film-forming substances of the coating. Currently, micro-arc oxidation of magnesium alloys is mostly carried out in conventional aqueous solutions and can be classified into silicate, phosphate, and aluminate systems based on the main electrolyte components [17,18,19]. The silicate system is currently the most suitable aqueous solution system for magnesium alloys. It can promote reactions under a wide range of electrolyte temperatures and current densities, forming coatings mainly composed of MgSiO_3_, Mg_2_SiO_4_, and MgO [15,20,21,22]. However, the coating formed by the aqueous solution system is mainly composed of MgO, which is prone to hydrolysis into Mg(OH)_2_ in corrosive media, resulting in volume expansion, structural damage, and the loss of coating adhesion and protective ability [23,24,25]. Therefore, additives are often introduced into basic electrolytes such as silicates to transform the main film-forming substances into more stable compounds like MgF_2_ or Mg_2_SiO_4_ [21,26,27,28].

Sun Le et al. [29] investigated the effect of Na_2_SiO_3_ in the electrolyte on the micro-arc oxidation of AZ91D magnesium alloy. The results showed that it could improve the integrity and flatness of the film surface. Zhang et al. [30] found that adding Na_2_SiO_3_ and SiO_3_^2^^−^ to the phytic acid electrolyte was beneficial for thickening the micro-arc oxide film layer and enhancing the corrosion resistance of AZ91HP magnesium alloy. In addition to traditional systems, new systems such as composite electrolytes and fluorine-containing electrolytes have emerged in recent years. Liu et al. [31] and Kazanski et al. [17] demonstrated that the addition of F^−^ to the electrolyte could enhance the compactness of the coating, promote its thickening, and improve corrosion resistance. The objective of this work is to elucidate the role of KF addition in the formation mechanism of magnesium silicate coatings in sodium silicate-based electrolytes. By analyzing phase composition, morphology, and corrosion behavior, we aim to clarify the synergistic effect of SiO_3_^2^^−^/F^−^ on enhancing the content of stable magnesium silicate compounds and to propose the underlying formation mechanism.

## 2. Experimental Section

### 2.1. Materials and Reagents

The base material used in this experiment was an AZ31A magnesium alloy sheet. The substrate remained in its as-received condition, with no subsequent heat treatment applied. The specific composition is shown in Table 1. The magnesium alloy plate was cut into square samples of 40 mm × 40 mm × 4 mm. Before the experiment, all the sample surfaces need to be pre-treated. The surfaces should be mechanically polished in sequence with 150–1500 mesh gradient sandpaper and then rinsed under running water and transferred to anhydrous ethanol for ultrasonic degreasing treatment (3 min). Finally, they should be purged and dried for later use. In the experiment, insulated wires were suspended in the electrolyte through the sample pretreatment holes. During the experiment, the anode was a magnesium alloy sheet and the cathode was a circular coil.

The basic electrolyte of this experiment was composed of sodium silicate (Na_2_SiO_3_·9H_2_O) and sodium hydroxide (NaOH). The concentration of sodium silicate in the basic electrolyte was varied, and a specific amount of potassium fluoride was added. The composition of the electrolyte is shown in Table 2. For the sake of brevity, the following designations refer to the samples prepared under the corresponding conditions. This experiment employed a constant current unipolar mode. The core parameters were as follows: current density, 2 A/dm^2^; frequency, 500 Hz; duty cycle, 20%; and processing time, 15 min. The adjustable ranges of the device parameters were frequency, 50–2000 Hz; pulse width, 10–500 μs; voltage, 0–750 V; and current, 0–30 A. A 3 L beaker served as the electrolytic cell, which was equipped with an internal 316 L stainless steel cathode coil, a circulating refrigeration system to maintain the temperature below 30 °C, and a magnetic stirring system to ensure uniform temperature and concentration.

### 2.2. Experimental Techniques

The morphology, structure, and composition of the coatings were characterized using X-ray diffraction (XRD) (Malvern Panalytical, Almelo, Netherlands) and scanning electron microscopy (SEM; JEOL JSM-7900F, Tokyo, Japan) equipped with an energy-dispersive X-ray spectroscopy (EDS; JEOL JSM-IT500A, Japan) system. The phase composition of the coatings was determined by XRD, while the coating thickness was measured with an eddy current thickness gauge. Potentiodynamic polarization curves were recorded using an Autolab PGSTAT 302N electrochemical workstation (Autolab PGSTAT 302N, Herisau, Switzerland) after the samples had been immersed for 1 h. Measurements were carried out in a conventional three-electrode configuration, using a saturated calomel electrode as the reference electrode and a platinum electrode as the counter electrode. The potential scan rate was 10 mV/s.

## 3. Results and Discussion

### 3.1. The Influence of SiO_3_ ^2−^/F^−^ on the Micro-Arc Oxidation Discharge Characteristics of Magnesium Alloy Surfaces

**Figure 1:**
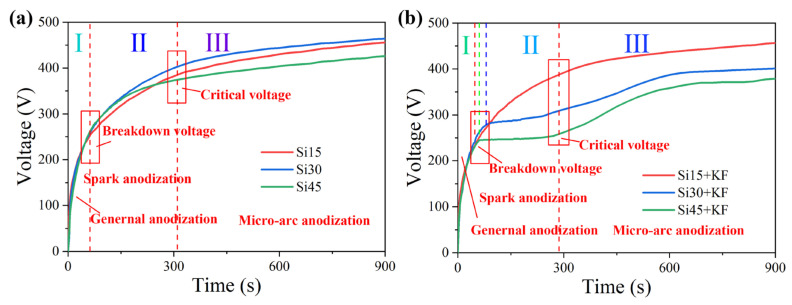
MAO time voltage curve of Mg alloy in different SiO_3_^2−^/F^−^ electrolyte: (**a**) Si; (**b**) Si + KF.

As shown in Figure 1, within the first 100 s, the voltage increases sharply during the anodic oxidation stage, and a passivation film forms on the surface of the metal substrate. Subsequently, silver-white sparks appear on the anode surface, indicating the breakdown of the oxide film. At this point, the micro-arc oxidation reaction enters the micro-arc discharge stage, and the voltage reaches the breakdown voltage. As can be seen in Figure 1a, with the increase in sodium silicate concentration, the breakdown voltages of samples Si 15, Si 30, and Si 45 are approximately 231, 235, and 241 V, respectively, indicating that an increase in sodium silicate concentration can reduce the breakdown voltage. By referring to Table 2, it can be concluded that due to the increase in sodium silicate concentration, the electrical conductivity of the electrolyte enhances, and the resistance value decreases. As shown in Figure 1, within the first 100 s, the voltage rises rapidly during the anodic oxidation stage, and a passivation film forms on the surface of the metal substrate. Subsequently, sparks appear on the anode surface, indicating the breakdown of the oxide film. At this point, the micro-arc oxidation reaction enters the micro-arc discharge stage, and the voltage reaches the breakdown voltage. As can be seen in Figure 1a, with the increase in sodium silicate concentration, the breakdown voltages of samples Si 15, Si 30, and Si 45 are approximately 231, 235, and 241 V, respectively, indicating that an increase in sodium silicate concentration increases the breakdown voltage. Referring to Table 2, it can be concluded that the increase in sodium silicate concentration enhances the electrical conductivity of the electrolyte and reduces its resistance. However, during the anodic oxidation stage, the coating also thickens, and a higher voltage is required for the passivation film to be punctured. In Figure 1b, as the concentration of sodium silicate increases, the breakdown voltages of samples Si 15+KF, Si 30+KF, and Si 45+KF are approximately 236, 270, and 241 V, respectively. Combined with Table 3, it can be seen that when the concentration of sodium silicate increases to 30 g/L, the addition of potassium fluoride enhances the conductivity of the electrolyte. However, it also accelerates the coating growth rate, which leads to an increase in coating thickness and a consequent rise in breakdown voltage. When the concentration of sodium silicate is raised to 45 g/L, the conductivity of the electrolyte is further enhanced, and its resistance decreases. However, the coating growth rate is limited, and the thickness does not increase proportionally; consequently, the breakdown voltage drops.

During the second stage, once the working voltage exceeds the critical threshold, dielectric breakdown occurs in the ceramic layer, accompanied by a significant intensification of discharge. The discharge color transitions from silvery-white to orange-yellow. Although the coating growth rate continues to rise, the voltage increases at a slower rate. In Figure 1a, the increase in the concentration of silicate in the electrolyte significantly enhanced the intensity of the plasma electrochemical reaction. When the electrolyte concentration rose to 45 g/L, due to the ion migration rate and reaction kinetics in the solution, although the anode film formation rate still maintained an upward trend, the electric field intensity required to maintain the discharge instead showed a downward trend. In Figure 1b, after the sodium silicate concentration was increased to 30 g/L and 45 g/L, the voltage did not continue to increase during the spark discharge stage after the oxide film was broken down. The increase in sodium silicate concentration and the addition of potassium fluoride led to an increase in the ion concentration in the electrolyte, making the breakdown phenomenon on the sample surface more intense and the discharge spark larger. At this point, the coating is constantly melting under intense spark discharges, and the growth rate of the coating is slow. As the micro-arc oxidation reaction continues, anions in the electrolyte keep moving towards the anode, promoting the continuous deposition of anions on the anode surface. However, the growth rate of the coating is hindered, and the generated coating compounds continuously melt into the electrolyte, causing the anode resistance to increase and the voltage to gradually rise slowly and stabilize.

**Figure 2:**
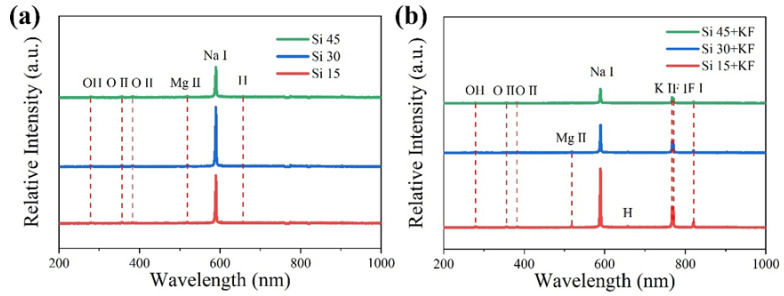
OES spectra of MAO of Mg alloys in different SiO_3_^2−^/F^−^ electrolytes for 15 min: (**a**) Si; (**b**) Si + KF.

Figure 2 shows the OES spectra of magnesium alloys after 15 min of micro-arc oxidation in different SiO_3_^2^^−^/F^−^ electrolytes. As shown in Figure 1, during the third stage of micro-arc oxidation, when the voltage stabilizes at its highest value, the discharge sparks were recorded spectrally. OES was used to capture the spark of micro-arc oxidation discharge within the wavelength range of 200 to 1000 nm, ensuring that the characteristic emission lines of the discharged species are within the monitoring range. Observation of Figure 2 reveals that the variation in discharge intensity, as measured by the Na I 589.46 nm spectral line, is consistent with the trend of the micro-arc oxidation termination voltage across different sodium silicate concentrations. In Figure 2a, in addition to the Na I emission line at 589.46 nm, spectral lines corresponding to Mg II at 519.40 nm, O II at 383.05 nm, O II at 391.19 nm, H at 656.41 nm, and OH at 285.05 nm were also observed, indicating the participation of these elements (in atomic or ionic form) in the micro-arc oxidation process. Among them, the presence of Na I 589.46 nm, O II 383.05 nm, O II 391.19 nm, and OH 285.05 nm suggests that ionic species from the electrolyte actively participated in the reaction. The strong Na I 589.46 nm peak indicates that electrolytes such as sodium silicate and sodium hydroxide were primarily consumed during the process. Meanwhile, the Mg II line at 519.40 nm indicates that, under intense discharge-induced breakdown, magnesium atoms from the substrate migrated to the surface and participated in the reaction. During the micro-arc oxidation process, an emission line at Hα 656.41 nm was observed, indicating that water in the electrolyte dissociated into atomic oxygen and hydrogen under the intense micro-arc discharge. As shown in Figure 2b, after the addition of potassium fluoride, strong spectral peaks corresponding to K I 767.11 nm, F I 771.13 nm, and F I 820.48 nm were detected in the optical emission spectroscopy (OES) results. This confirmed that potassium fluoride participated in the micro-arc oxidation reaction at the anode and enhanced the intensity of the anodic spark discharge.

### 3.2. Effects of SiO_3_^2−^/F^−^ on the Phase Composition and Microstructure of Micro-Arc Oxidation Coatings on Magnesium Alloys

**Figure 3:**
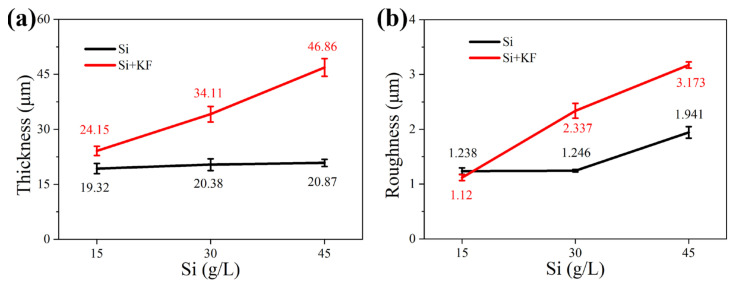
Thickness and roughness of MAO coating of Mg alloy in different SiO_3_^2−^/F^−^ electrolyte: (**a**) thickness; (**b**) roughness.

Figure 3 shows the thickness and roughness of the micro-arc oxidation coating on magnesium alloys in different SiO_3_^2^^−^/F^−^ electrolytes. In Figure 3a, when potassium fluoride is not added, as the concentration of sodium silicate increases from 15 g/L to 45 g/L, the coating thickness only increases from 19.32 μm to 20.81 μm. This indicates that increasing the sodium silicate concentration alone does not significantly increase the thickness, as the limited reaction between silicate ions and magnesium ions restricts coating growth. After the addition of potassium fluoride, the thickness increases significantly, and a higher sodium silicate concentration further accelerates this growth. This is because F^−^ promotes the co-deposition of silicate ions, hydroxide ions, and F^−^ itself with magnesium ions to form the coating. In Figure 3b, the coating roughness generally increases with thickness. However, the roughness of Si 45 (45 g/L sodium silicate) is significantly higher than that of Si 15 and Si 30, which have comparable thicknesses. This is because an excessively high concentration of silicate ions intensifies the discharge. Under these conditions, some silicate ions do not fully participate in film formation but instead contribute to excessive discharge around the channels. This causes slight surface ablation of the coating, reducing its flatness and thereby increasing its roughness.

**Figure 4:**
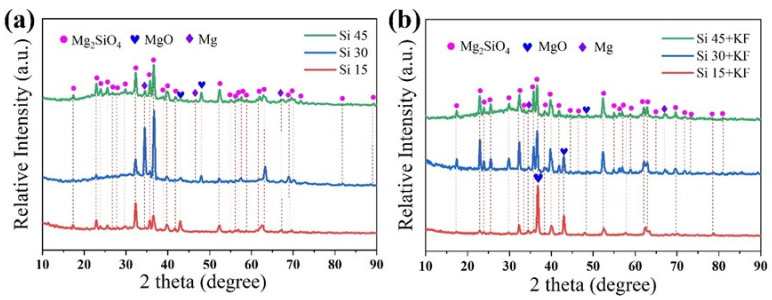
XRD pattern of MAO coating of Mg alloy in different SiO_3_^2−^/F^−^ electrolytes: (**a**) Si; (**b**) Si + KF.

Figure 4 shows the XRD patterns of micro-arc oxidation coatings on magnesium alloys in different SiO_3_^2^^−^/F^−^ electrolytes. The main phases of the coating are Mg_2_SiO_4_ and MgO, while the Mg peak originates from the substrate. There are two pathways for the formation of MgO: (1) OH^−^ in the electrolyte migrates to the anode and combines with Mg^2^^+^ to form Mg(OH)_2_, which is then dehydrated under high temperature and high pressure during discharge to form MgO. (2) The reactive oxygen species produced by strong ionization during the discharge process combine with Mg^2^^+^ to form MgO. Experiments confirmed that Mg_2_SiO_4_ is formed through the following pathways: (1) SiO_3_^2^^−^ electromigrates to the anode interface, where it reacts with activated magnesium via solid-phase diffusion under the high-temperature conditions of the plasma (10^3^–10^4^ K), forming a spinel-structured Mg_2_SiO_4_ phase. (2) Within the discharge micropores, SiO_3_^2^^−^ dissociates into SiO_2_ precursors, which subsequently undergo solid-phase sintering with molten MgO to form Mg_2_SiO_4_. Figure 4a shows that with the increase in sodium silicate concentration, the peak intensity of Mg_2_SiO_4_ first increases and then decreases, while the peak intensity of MgO slightly increases. This trend indicates that an appropriate concentration increases the Mg_2_SiO_4_ content, but an excessively high concentration becomes unfavorable. The increase in OH^−^ concentration is conducive to the formation of MgO. This deviation from the solubility product rule [32] may be related to the hindrance of ion migration by the Mg_2_SiO_4_ layer. The Mg peak for the Si 30 sample is significantly more intense, which might be due to the presence of large discharge channels and a thinner coating in the XRD scanning area. Figure 4b shows that after the addition of potassium fluoride, with the increase in sodium silicate concentration, the peak intensity of Mg_2_SiO_4_ still increases first and then decreases, the peak intensity of MgO continues to decrease, and there is no MgF_2_ peak. This is related to the solubility product (Ksp) of Mg_2_SiO_4_, MgF_2_, and Mg(OH)_2_: as the concentrations of SiO_3_^2^^−^ and OH^−^ increase, Mg(OH)_2_ and Mg_2_SiO_4_ are preferentially formed. Compared with the fluorine-free system, when sodium silicate is 15 g/L, potassium fluoride increases the concentration of OH^−^, while that of SiO_3_^2^^−^ is lower, which is conducive to the formation of MgO. Potassium fluoride enhances the electrical conductivity and increases the OH^−^ concentration, thereby facilitating faster migration of OH^−^ to the anode to form MgO. As the concentration of SiO_3_^2^^−^ further increases, the F^−^ synergistic effect promotes the deposition of SiO_3_^2^^−^ to form Mg_2_SiO_4_. No MgF_2_ peak was detected because its Ksp is higher than that of Mg_2_SiO_4_ and Mg(OH)_2_, resulting in an excessively low content, or because it exists in an amorphous form.

**Figure 5:**
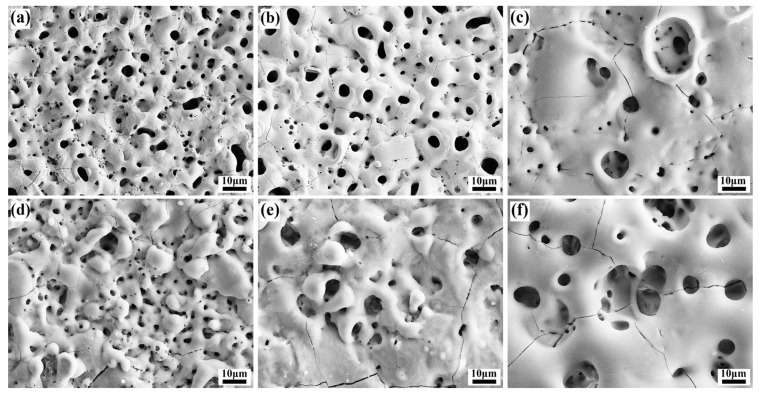
Surface morphologies of MAO coatings on Mg alloys in different SiO_3_^2−^/F^−^ electrolytes: (**a**–**c**) Si 15-Si 30-Si45; (**d**–**f**) Si 15 + KF-Si 30 + KF-Si 45 + KF.

Figure 5 shows the surface morphology of micro-arc oxidation coatings on magnesium alloys in different SiO_3_^2^^−^/F^−^ electrolytes. The surface of the coating is uneven, with a large number of micro-holes, cracks, and particles of different sizes distributed around the holes. Micropores originate from the discharge channels formed when the plasma discharge breaks through the coating during the micro-arc oxidation process. The particles are solidified from molten oxides that splash out of the discharge channel under instantaneous high temperature and pressure and then rapidly cool upon contact with the cold electrolyte, ultimately depositing around the pores. Cracks are generated by the thermal stress concentration resulting from the localized action of discharge sparks. With reference to Figure 5a–c and Table 3, as the sodium silicate concentration increases, the average pore size on the coating surface increases, the porosity slightly decreases, and the number of molten oxide particles around the pores is reduced. This is attributed to the increased accumulation of SiO_3_^2^^−^ around the discharge channel, enhancing the plasma discharge. In Figure 5a, the discharge is mild during the middle and later stages, allowing the molten material to easily fill the channels or deposit on the surface, forming gentle protrusions around the pores. In Figure 5c, the discharge is intense, and SiO_3_^2^^−^ is more likely to form a film with Mg^2^^+^ within the channel. Under the higher pressure, it becomes difficult for the molten material to fill the channels and deposit on the surface, resulting in a reduction in particle size. The concentration of sparks leads to an increase in microcracks. The molten material promotes the merging of adjacent holes, leading to an increase in their size, while the protrusions around the holes become more pronounced, corresponding to the increase in roughness as shown in Figure 3b. Figure 5d–f show that compared with the potassium-free coating, the surface oxide particles decrease after adding potassium fluoride, indicating that more molten material fills the channels or is remelted and sintered in the coating after splashing. This observation is consistent with the increase in thickness shown in Figure 3a. With reference to Figure 3a and Table 4, the coating thickness increases after adding potassium fluoride, while the porosity and average pore size decrease. This is because F^−^ promotes the co-deposition of SiO_3_^2^^−^, OH^−^, and Mg^2^^+^ to form the coating, while the molten material simultaneously fills the discharge channels, thereby reducing porosity. As the concentration of sodium silicate increases, the surface in Figure 5f is smoother, without obvious protrusions, and the molten material appears denser than that in Figure 5d. The increased concentration enhances the electrical conductivity, leading to a higher coating partial pressure, more intense plasma discharge, and elevated surface temperature. These factors collectively improve the fluidity of the molten material, allowing it to distribute uniformly around the pores during sintering. In Table 4, the average pore size of Si 30 + KF is lower than that of Si 15 + KF because more molten material fills the channels, preventing the pores from connecting and growing.

**Figure 6:**
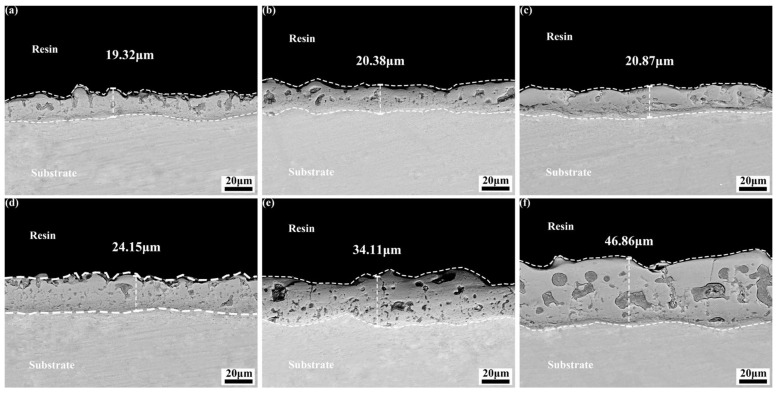
Cross-sectional morphologies of MAO coatings on magnesium alloys prepared in different electrolytes: (**a**–**c**) coatings from electrolytes with 15, 30, and 45 g/L Na_2_SiO_3_ without KF; (**d**–**f**) coatings from electrolytes with the same silicate concentrations and KF addition.

Figure 6 shows the cross-sectional morphologies of micro-arc oxidation coatings on magnesium alloys in different SiO_3_^2^^−^/F^−^ electrolytes. Figure 6a–c show that increasing the concentration of sodium silicate does not significantly increase the coating thickness. Due to the increased SiO_3_^2^^−^ concentration, its migration toward the anode intensifies, raising the reaction energy. This causes the molten oxide to partially fill the discharge channels and pores, while another portion is removed by the electrolyte. (c) As shown in the figure, the reduction in external pores and the filling of channels hinder the reaction between SiO_3_^2^^−^ and Mg^2^^+^ for film formation. Figure 6d–f indicate that after adding an appropriate amount of potassium fluoride, increasing the concentration of sodium silicate continuously increases the coating thickness and enhances its compactness. Potassium fluoride increases the number of discharge channels and intensifies the reaction, enabling more SiO_3_^2^^−^ to participate in film formation, while the generated gas becomes trapped within the coating. The pores originate from gas trapped by the high temperature and pressure or blocked by the rapid solidification of the molten material during cooling. Potassium fluoride and sodium silicate exhibit a strong synergistic interaction, which significantly promotes the deposition of SiO_3_^2^^−^ and increases the coating thickness.

**Figure 7:**
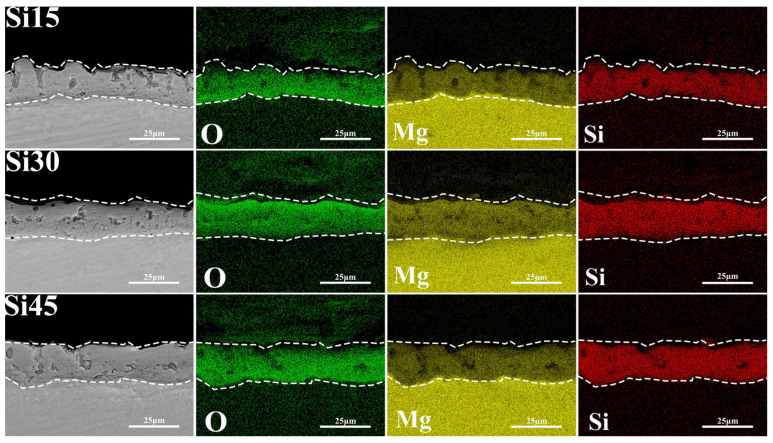
Cross-sectional morphologies and corresponding element distributions of magnesium alloy MAO coatings under different SiO_3_^2−^/F^−^ electrolytes: Si15, Si30, Si45.

Based on the analysis of Figure 7 and Table 5, the promoting effect of potassium fluoride on fluoride formation is limited, and the fluorine element is mainly enriched on the inner side of the coating. This distribution characteristic is jointly determined by the electronegativity of anions in the electrolyte, the ionic radius, and the solubility product of MgF_2_. Among them, the F^−^ ion has the smallest radius, which is 0.126 nm. Under the action of an electric field, it is more likely to migrate towards the anode, resulting in a higher fluorine content inside the coating. The data in Table 5 show that in the samples with low sodium silicate concentration and added potassium fluoride, the silicon content is relatively low. Combined with the observation in Figure 4b, the main film-forming substance of the coating is magnesium oxide. This indicates that under low-sodium silicate conditions, the addition of potassium fluoride is conducive to the formation of magnesium oxide. The mechanism of action lies in that potassium fluoride not only increases the concentration of OH^−^ in the electrolyte but also enhances the plasma discharge process, raising the concentration of O^2^^−^ produced by the ionization of SiO_3_^2^^−^, OH^−^, and H_2_O, and promoting the formation of magnesium oxide. As the concentration of sodium silicate increases, the content of fluorine in the coating decreases, while the contents of silicon and oxygen increase, and the main film-forming substance transforms into magnesium silicate. Potassium fluoride effectively promoted the participation of OH^−^ and SiO_3_^2^^−^ in the micro-arc oxidation reaction. The decrease in fluorine content is closely related to the solubility product of Mg(OH)_2_, MgF_2_, and Mg_2_SiO_4_. The solubility product of MgF_2_ is 5.16 × 10^−^^11^, that of Mg(OH)_2_ is 5.61 × 10^−^^12^, while the solubility of Mg_2_SiO_4_ is extremely low, with its solubility product constant Ksp value approximately ranging from 10–20 to 10–25. Because the solubility product of Mg_2_SiO_4_ is much smaller than that of MgF_2_ and Mg(OH)_2_, and the concentration of SiO_3_^2^^−^ ions in the electrolyte is relatively high, magnesium silicate is more likely to form during the micro-arc oxidation process.

### 3.3. Influence of SiO_3_^2−^/F^−^ on the Corrosion Protection Performance of Micro-Arc Oxidation Coatings on Magnesium Alloys

**Figure 8:**
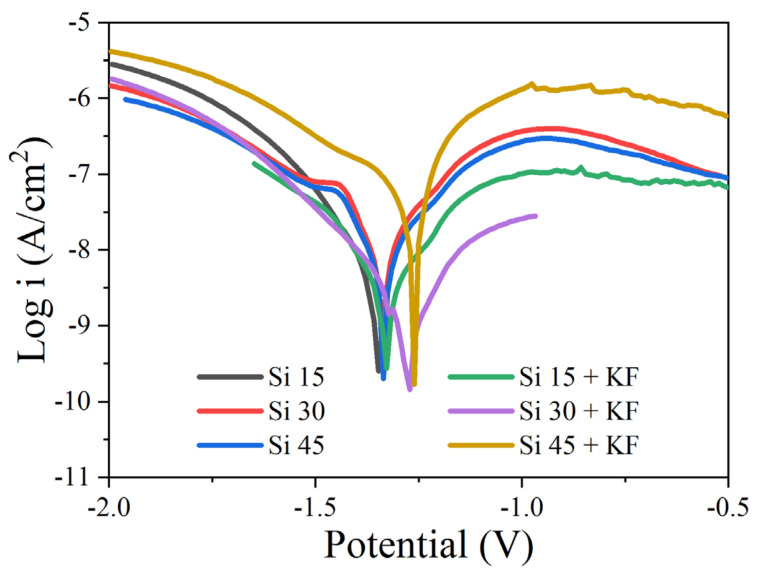
Potentiodynamic polarization curves of MAO coatings on Mg alloy in NaCl solution prepared in different SiO_3_^2−^/F^−^ electrolytes.

Figure 8 presents the potentiodynamic polarization curves of micro-arc oxidation (MAO) coatings formed on magnesium alloys in various SiO_3_^2^^−^/F^−^ electrolytes, with the corresponding fitting parameters summarized in Table 6. Analysis of the polarization behavior and derived corrosion parameters reveals that KF addition exerts a pronounced inhibiting effect on the corrosion of samples prepared in low-silicate electrolytes (Si 15/30), whereas it leads to a deterioration in corrosion performance for the high-silicate sample (Si 45). In the absence of potassium fluoride, increasing the concentration of sodium silicate in the electrolyte results in a more noble corrosion potential and a lower corrosion current density. This trend is attributed to the increased formation of magnesium silicate within the coating matrix, which enhances coating stability as its content rises. Upon the addition of potassium fluoride, an increase in sodium silicate concentration results in a more noble corrosion potential of the coating. However, the corrosion current density initially decreases and subsequently increases. This behavior is attributed to the relatively large surface pore size and the significant internal defects observed in the Si 45 + KF coating, which collectively contribute to a slight reduction in corrosion resistance. This can be attributed to the high silicate concentration inducing localized defects within the coating, thereby promoting localized corrosion.

## 4. Discussion

**Figure 9:**
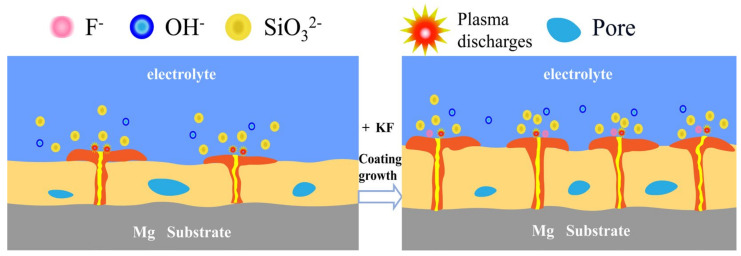
Schematic diagram of coating formation mechanism of MAO coatings on Mg alloys in different SiO_3_^2−^/F^−^ electrolytes.

Figure 9 schematically illustrates the film-forming mechanism of magnesium silicate coatings produced via micro-arc oxidation (MAO) on magnesium alloys under varying concentrations of SiO_3_^2^^−^ and F^−^. As shown in Figure 1 and Figure 3, the addition of potassium fluoride led to an increase in both the pH and the electrical conductivity of the electrolyte, while the working voltage slightly decreased. When the sodium silicate concentration is 15 g/L, and potassium fluoride is introduced, F^−^ ions—due to their high electronegativity—readily migrate toward the anode during the anodic oxidation stage, facilitating the formation of a passivation film. After the plasma-induced breakdown of this film, additional discharge channels are formed. Both OH^−^ and F^−^ ions from the electrolyte participate in the film-forming process, resulting in increased coating thickness. This phenomenon is consistent with the observation results of Thanaa et al. [18] in fluorine-containing electrolytes. With the sodium silicate concentration raised to 30 g/L and 45 g/L, data in Figure 4 and Table 4 and Table 5 indicate a continuous increase in the magnesium silicate content within the coating. However, in the absence of potassium fluoride, the coating thickness remains largely unchanged. This is attributed to the higher SiO_3_^2^^−^ concentration intensifying the plasma discharge reaction on the anode surface, leading to the continuous dissolution of the coating material and thereby inhibiting its growth.

The addition of potassium fluoride further enhances electrical conductivity. During anodic oxidation, F^−^ promotes the formation of a denser passivation film, and the plasma discharge becomes more vigorous. During the spark discharge stage, the working voltage increases at a slower rate due to coating dissolution. As the MAO reaction proceeds, the plasma generates more discharge channels, allowing more SiO_3_^2^^−^ to enter these channels and participate in film formation. This process enhances the deposition of silicate compounds, thereby increasing both the Mg_2_SiO_4_ content and the overall coating thickness. This is in sharp contrast to the findings of Zhang et al. [26] in a single silicate system, where their coating was mainly composed of MgO. In the SiO_3_^2−^/F^−^ composite electrolyte of this work, the main phase of the coating successfully transformed into the more stable Mg_2_SiO_4_. This fundamental difference in phase composition confirms that the introduction of F^−^ not only enhances the conductivity but also its core role lies in generating a synergistic effect with SiO_3_^2^^−^, by altering the discharge characteristics and providing thermodynamic driving force (extremely low solubility product of Mg_2_SiO_4_), jointly guiding and promoting the preferential formation and deposition of magnesium silicate ceramic layers. As evidenced by Figure 5 and Table 4, the rapid film formation rate and intense plasma discharge lead to the formation of larger discharge pores. When molten oxides are ejected onto the coating surface, they are insufficient to completely fill the discharge channels, resulting in increased surface porosity and larger pore sizes.

## 5. Conclusions

1. Increasing the concentration of sodium silicate or adding KF can both enhance the conductivity and pH of the electrolyte. When KF was not added, increasing the concentration of sodium silicate enhance plasma discharge, but the dissolution rate of the coating was similar to the growth rate, so there was no significant increase in thickness. After adding KF, the number of discharge channels increased, the growth rate of the coating accelerated, and the thickness continued to increase with the increase in sodium silicate concentration.

2. As the concentration of sodium silicate in the electrolyte increased, the silicon content in the coating increased, and its main phase changed from MgO to Mg_2_SiO_4_. The addition of KF facilitated this transformation process. When KF was not added, the pore size of the coating surface increased with the increase in sodium silicate concentration, the porosity slightly decreased, and the corrosion resistance slowly improved.

3. After adding KF, both the porosity and pore size of the coating were significantly reduced. With the increase in sodium silicate concentration, the pore size showed a trend of first decreasing and then increasing, while the porosity continued to decline. Among them, the Si 30 + KF sample had the fewest surface defects and demonstrated the best corrosion resistance.

## Figures and Tables

**Table 1:** Compositions of AZ31A Mg alloy (wt.%).

Element	Al	Zn	Si	Mn	Fe	Cu	Mg
**Contents (%)**	3.12	1.04	0.006	0.44	0.001	0.001	bal.

**Table 2:** Composition of the SiO_3_^2−^/F^−^ electrolytes.

Specimens	Electrolyte
Si 15	15 g/L Na_2_SiO_3_·9H_2_O + 5 g/L NaOH
Si 30	30 g/L Na_2_SiO_3_·9H_2_O + 5 g/L NaOH
Si 45	45 g/L Na_2_SiO_3_·9H_2_O + 5 g/L NaOH
Si 15 + KF	15 g/L Na_2_SiO_3_·9H_2_O + 5 g/L NaOH + 0.129 mol/L KF·2H_2_O
Si 30 + KF	30 g/L Na_2_SiO_3_·9H_2_O + 5 g/L NaOH + 0.129 mol/L KF·2H_2_O
Si 45 + KF	45 g/L Na_2_SiO_3_·9H_2_O + 5 g/L NaOH + 0.129 mol/L KF·2H_2_O

**Table 3:** Basic properties of the electrolyte.

Specimens	pH	Conductivity (mS/cm)
Si 15	12.8	40.6
Si 30	13.02	52.4
Si 45	13.18	63.1
Si 15+KF	13.11	50.9
Si 30+KF	13.31	59.3
Si 45+KF	13.39	70.5

**Table 4:** Surface porosity and average size of the MAO coating of Mg alloy in different SiO_3_^2−^/F^−^ electrolytes.

Specimens	Average Size (μm)	Area (%)
Si 15	1.487	8.732
Si 30	2.14	8.632
Si 45	3.817	8.616
Si 15 + KF	1.418	8.053
Si 30 + KF	1.209	7.83
Si 45 + KF	3.529	7.438

**Table 5:** Elemental content of MAO coatings on Mg alloy in different SiO_3_^2−^/F^−^ electrolytes.

Specimens	Content of Elements (wt.%)					
	Mg	O	Si	F	Na	K
Si 15 + KF	38.19	41.94	13.04	5.26	1.28	0.29
Si 30 + KF	29.90	43.48	18.48	4.02	3.13	0.99
Si 45 + KF	22.99	44.25	22.92	1.05	6.67	2.13

**Table 6:** Polarization curve parameters of MAO coatings on Mg alloy in different SiO_3_^2−^/F^−^ electrolytes.

Specimens	*E_corr_* (mV vs. Ag/AgCl)	*i_corr_* (A/cm^2^)
Si 15	−1357	5.13 × 10^−8^
Si 30	−1336	4.76 × 10^−8^
Si 45	−1328	3.62 × 10^−8^
Si 15 + KF	−1327	8.91 × 10^−9^
Si 30 + KF	−1276	3.89 × 10^−9^
Si 45 + KF	−1268	9.12 × 10^−8^

## Data Availability

The original contributions presented in this study are included in the article. Further inquiries can be directed to the corresponding author.
